# 腺相关病毒基因修饰后间充质干细胞来源的外泌体免疫调节功能的研究

**DOI:** 10.3760/cma.j.issn.0253-2727.2021.06.003

**Published:** 2021-06

**Authors:** 丽 刘, 文威 邵, 成文 李, 四洲 冯, 晓磊 裴

**Affiliations:** 1 中国医学科学院血液病医院、北京协和医学院（中国医学科学院血液学研究所），实验血液学国家重点实验室，国家血液系统疾病临床医学研究中心，天津 300020 State Key Laboratory of Experimental Hematology, National Clinical Research Center for Blood Diseases, Institute of Hematology & Blood Diseases Hospital, Chinese Academy of Medical Sciences & Peking Union Medical College, Tianjin 300020, China; 2 天津大学，医学工程与转化医学研究院 300072 Academy of Medical Engineering and Translational Medicine, Medical College, Tianjin University, Tianjin 300072, China; 3 北卡罗莱纳大学（教堂山），医学院基因治疗中心，北卡罗莱纳州，美国 Gene Therapy Center, University of North Carolina at Chapel Hill, NC 27599, USA

**Keywords:** 间充质干细胞, 外泌体, 移植物抗宿主病, 腺相关病毒, 基因修饰, Mesenchymal stem cells, Exosome, Graft-versus-host disease, Adeno-associated virus, Genetic modification

## Abstract

**目的:**

验证天然状态间充质干细胞来源的外泌体（MSC-exosome）治疗小鼠急性移植物抗宿主病（GVHD）的效果和可能机制，探索并建立定向基因修饰MSC-exosome的方法并验证修饰后MSC-exosome的功能。

**方法:**

构建小鼠急性GVHD模型，观察比较不同剂量MSC-exosome组和MSC组的生理指标评分、生存和体重减低程度；进而通过体外T细胞活化实验和体内OVA抗原特异性T细胞活化实验检测比较组间活化T细胞的增殖水平。构建重组表达载体，获得携带PD-L1和PD-L1-ITGB1的AAV2YF3突变体，进而感染人MSC，分离获得其外泌体。检测比较天然状态和修饰后MSC-exosome在体外和体内对活化T细胞的增殖水平和调节性T细胞（Treg）比例的影响。

**结果:**

①MSC-exosome（300 µg×3次）和小鼠MSC（1×10^6^×3次）均能够有效改善急性GVHD小鼠的生理指标评分、生存和体重减低程度。②相比IL-2对照组，10、25、50 µg人MSC-exosome和1×10^6^人MSC体外共孵育处理均能够抑制活化T细胞的增殖，增殖比例分别为86.0％（IL-2）、40.0％、39.6％、42.8％和41.0％；相比PBS对照组，50、100、200 µg小鼠MSC-exosome和1×10^6^小鼠MSC在体内均能够抑制抗原特异性活化的OT-1细胞的增殖，增殖比例分别为42.6％、33.1％、14.2％、10.6％和14.6％。③携带PD-L1和PD-L1-ITGB1的AAV2YF3突变体定向修饰人MSC-exosome的表达率分别超过40％和60％。④相比天然状态，PD-L1和PD-L1-ITGB1定向修饰后MSC-exosome在体内对抗原特异性活化的OT-1细胞具有更好的增殖抑制效果；在体外亦能明显抑制活化T细胞的增殖，并诱导提高Treg的比例。

**结论:**

MSC-exosome具有与MSC相似的免疫调节作用。经过PD-L1和PD-L1-ITGB1修饰后的MSC-exosome能够有效抑制活化T细胞的增殖，并且能够诱导提高Treg的比例。

## Abstract (en)

**Objective:**

To verify the effects and mechanisms of natural MSC-exosome in treating acute GVHD in mice, explore and establish a method for targeted modification of MSC-exosome, and verify the functions of the modified MSC-exosome.

**Methods:**

In different doses of MSC-exosome groups and MSC group, weight loss in acute GVHD mice was observed; then the proliferation levels of activated T cells were measured through T cell activation experiment in vitro and OVA antigen-specific T cell activation experiment in vivo. AAV2YF3 mutants carrying PD-L1 and PD-L1-ITGB1 were obtained after the construction of recombinant expression vectors and were then applied to infect human MSC to modify their exosome. The immunoregulatory functions of the modified MSC-exosome were measured with the abovementioned methods.

**Results:**

①Mouse MSC-exosome（300 µg × 3 times）and MSC（1 × 10^6^ × 3 times）effectively alleviated the weight loss in acute GVHD mice. ②Compared with IL-2, 10, 25 and 50 µg human MSC-exosome inhibited the proliferation of activated T cells in vitro, respectively, 86.0％（IL-2）, 40.0％, 39.6％, and 42.8％; compared with PBS, 50, 100 and 200 µg mouse MSC-exosome inhibited the proliferation of antigen-specific activated OT-1 cells in vivo, respectively, 42.6％, 33.1％, 14.2％, and 10.6％. ③ After the infection of AAV2YF3 mutant carrying PD-L1 or PD-L1-ITGB1, the positive proportion of MSC-exosome exceeds 40％ and 60％, respectively. ④Compared with the natural state, MSC-exosome modified by PD-L1 or PD-L1-ITGB1 showed better proliferation inhibitory effect in vivo and increased the proportion of Treg cells in vitro.

**Conclusion:**

MSC-exosome exhibited similar immunomodulatory effects with MSC. MSC-exosome after PD-L1 and PD-L1-ITGB1-targeted modification effectively inhibited the proliferation of activated T cells and increased the proportion of Treg cells.

间充质干细胞（MSC）是一类具有多向分化潜能的前体细胞，不仅保留有自我更新能力，还具有免疫调节和促进组织修复等作用[1]–[2]，已经成为细胞治疗的重要来源之一。近年来，一些研究发现MSC来源的外泌体（MSC-exosome）具有与MSC相似的生物学作用，有望替代MSC细胞治疗[3]–[4]，MSC-exosome的应用已经成为新的研究热点。

外泌体是一种由活细胞分泌的膜性囊泡，直径40～150 nm，广泛存在于生物体体液中，参与细胞信号转导等生物活动[5]。在形成过程中，由于细胞来源、细胞状态和微环境的不同，不同的脂质、蛋白质、RNA或DNA被装载入外泌体中[5]。

相较于其来源的细胞，外泌体更易被大量获取，具有更高的安全性、低免疫原性和低成瘤性，并且不容易滞留在肺微循环，能够更充分地渗透入组织中以发挥作用[4],[6]。此外，MSC-exosome也是运输治疗性基因、药物、酶或RNA的理想形式，不仅能够保护其运载物免于被降解，并且能够辅助内吞作用以确保靶细胞摄取[5]。而且，与MSC类似，MSC-exosome膜具有可修饰性，能够被定向增强其特异的靶向性[7]。然而，目前对外泌体的认识还比较粗浅，经典的外泌体分离方法获得的外泌体异质性较高，如果通过基因修饰技术使外泌体上富集功能特异性蛋白，一方面能降低外泌体的异质性，另一方面也能有效地提高外泌体的功能性。因此，我们在本研究中验证了天然状态MSC-exosome在治疗小鼠急性移植物抗宿主病（GVHD）中的效果和可能机制，同时寻找到一种对MSC具有较好感染能力的腺相关病毒的突变体（AAV2YF3），并利用携带PD-L1和PD-L1-ITGB1的AAV2YF3对MSC-exosome进行基因修饰，最终发现修饰后MSC-exosome能够有效抑制活化T细胞的增殖，并诱导提高调节性T细胞（Treg）的比例。

## 材料与方法

一、细胞、抗体和试剂

1. 细胞、质粒和AAV：脐带血来源的人MSC（ATCC PCS-500-010）和HEK293细胞系（ATCC CRL-1573）购于美国ATCC公司，小鼠MSC（Strain C57BL/6 Mouse Mesenchymal Stem Cells，MUBMX-01001）购于美国Cyagen公司。人外周血单个核细胞（PBMC）来自于健康志愿者知情同意后自愿捐献。H2KB质粒、AAV病毒和慢病毒的包装质粒均来自于北卡罗莱纳大学李成文课题组[8]。

2. 抗体和试剂：X-VIVO™10（Lonza，瑞士），乳胶微球（Thermo Fisher，美国），APC抗小鼠或人CD9抗体、PE抗小鼠或人CD63抗体、纯化抗小鼠CD63抗体、羧基荧光素二醋酸盐琥珀酰亚胺酯（CFSE）细胞分裂检测试剂盒、重组人IL-2、APC抗小鼠或人CD4抗体、FITC抗人CD25抗体、PE抗人FOXP3抗体、APC抗小鼠或人CD8抗体、APC抗人PD-L1抗体、PE-Cy7抗小鼠CD69抗体（Biolegend，美国），纯化抗小鼠CD9抗体（eBioscience，美国），CD9单克隆抗体，克隆号VJ1/20（Abnova，美国），CD3功能性抗体（OKT-3）（PeproTech，美国），外泌体提取纯化试剂盒、BCA蛋白定量试剂盒（Thermofisher，美国），人CD3磁珠（Miltenyi Biotec，德国）。

二、实验动物

健康雌性C57BL/6及健康雄性BALB/c小鼠，6～8周龄，体重（20±3）g，购于北京华阜康生物科技有限公司；健康雌性OT-1小鼠，6～8周龄，体重（20±3）g，购于杰克森实验室。所有小鼠均饲养于SPF环境，所有动物实验均按照动物伦理委员要求操作。

三、急性GVHD小鼠模型的构建

参照文献[9]–[10]方法构建急性GVHD小鼠模型并进行状态评分。

四、外泌体基因修饰、纯化和鉴定

AAV的包装和纯化方法步骤在我们前期的报道中有详细描述[11]。AAV在体外感染MSC时的感染复数（MOI）为1000。感染72 h后，检测AAV携带基因的表达，收集细胞培养上清并提取外泌体。

收集MSC无血清培养上清，4 °C 300×*g*离心10 min以去除细胞，将离心后的上清转移至新的离心管中，4 °C 2000×*g*离心10 min以去除死细胞，将离心后的上清转移至新的离心管中，4 °C 10 000×*g*离心30 min以去除细胞碎片，将离心后的上清转移至新的超速离心管中，4 °C 100 000×*g*离心70 min，留沉淀，加入35 ml无菌PBS重悬，4 °C 100 000×*g*离心70 min，留沉淀，1 ml无菌PBS重悬沉淀后使用BCA法进行蛋白定量。以每管50 µl分装后−80 °C保存，备后续实验使用。

获得纯化外泌体后，Western blot鉴定外泌体的纯度，抗CD9（1∶1000稀释）和抗CD63（1∶1000稀释）抗体检测外泌体溶液中CD9和CD63的含量，抗β-actin（1∶1000稀释）抗体检测外泌体溶液中其他蛋白成分的污染状况，使用微球吸附方法检测外泌体溶液中CD9和CD63的表达量。详细过程参考前期研究报道[12]。

五、体外T细胞活化实验

密度梯度离心法分离得到PBMC，经偶联抗CD3抗体的磁珠分选得到其中的T淋巴细胞，使用CFSE染色后加入100 U/ml IL-2和100 U/ml抗CD3抗体（OKT-3）进行刺激，后均分至24孔板中，每孔细胞数为1×10^6^；每3孔为1组，分别加入PBS，人MSC（每孔1×10^5^），10、25、50 µl MSC-exosome溶液（1 µg/µl）；置于37 °C、5％ CO_2_、完全饱和湿度的培养箱中培养3 d后，上流式细胞仪检测CFSE水平，推算活化T细胞的增殖水平。

六、体内OVA抗原特异性T细胞活化实验和体外OT-1细胞早期活化实验参照文献[8]。

七、体外Treg的诱导分化实验

密度梯度离心法分离得到PBMC，经偶联抗CD3抗体的磁珠分选得到其中的T淋巴细胞，体外抗CD3（OKT-3，100 U/ml）+IL-2 100 U/ml+DMEM+10％FBS培养3 d，后均分至24孔板中，每孔500 µl；每3孔为1组，PBS、50 µl MSC-exosome、50 µl PDL1-MSC-exosome和50 µl PD-L1-ITGB1-MSC-exosome溶液（1 µg/µl）；置于37 °C、5％ CO_2_、完全饱和湿度的培养箱中培养5 d后，上流式细胞仪检测CD4^+^CD25^+^FOXP3^+^ Treg在CD4^+^ T细胞中的比例。

八、统计学处理

所有实验数据以均数±标准差表示，采用Student's *t*检验比较2个独立实验组的差异，*P*<0.05为差异有统计学意义。

## 结果

一、天然状态MSC-exosome能够有效治疗小鼠急性GVHD

超过90％的乳胶微球可以捕获MSC分泌的外泌体，而在对照新鲜培养基中仅为1.43％（图1A）。同时，Western blot也可以检测到MSC-exosome表达的CD9和CD63（图1B）。如图1C～E所示，建立急性GVHD小鼠模型，在+7、+10、+13 d分别经内眦静脉注射1×10^6^ MSC或300 µg MSC-exosome能有效改善急性GVHD小鼠的生理指标评分、生存和体重减低程度，100 µg和50 µg MSC-exosome处理组与PBS组相比有一定程度的改善，但差异无统计学意义。以上结果提示，MSC-exosome用于治疗小鼠急性GVHD时发挥了与MSC相似的免疫调节作用。

**图1:**
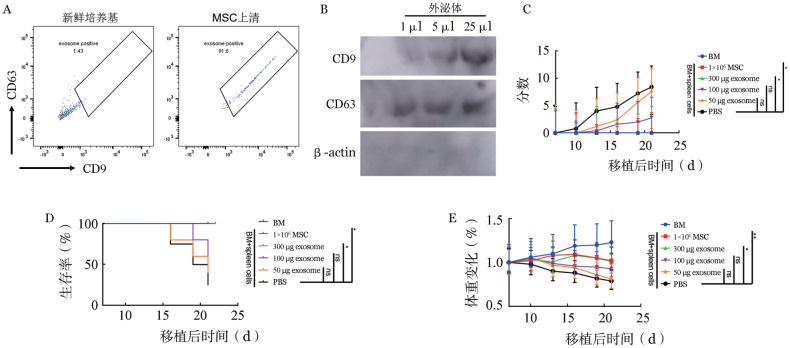
间充质干细胞（MSC）培养上清中外泌体的检测和对移植物抗宿主病（GVHD）的功能验证 A：使用乳胶微球吸附新鲜培养基或小鼠MSC培养上清中的蛋白后，标记抗小鼠CD9和抗小鼠CD63抗体，使用流式细胞仪检测外泌体表面CD9和CD63的表达；B：使用外泌体纯化试剂盒纯化获得新鲜培养基或小鼠MSC培养上清中的外泌体，将外泌体溶液浓度调整为1 µg/µl，Western blot检测新鲜培养基，1、5和25 µl外泌体溶液中CD9、CD63和内参对照β-actin的表达；C～E：构建小鼠急性GVHD模型，分别在+7、+10、+13 d分别内眦静脉注射PBS，1×10^6^ MSC，50、100、300 µg MSC-exosome，监测小鼠的生理指标评分（C）、生存状况（D）和体重减低程度（E）的变化（每组5只小鼠；ns：*P>0.05，*P<0.05；**P<0.01）

二、天然状态MSC-exosome在体外和体内对活化T细胞的抑制作用

如图2A所示，1×10^6^ CFSE标记的人外周血CD3^+^ T细胞，经过抗CD3（OKT-3，100 U/ml）+IL-2（100 U/ml）活化后，分别与1×10^5^人MSC，10、25、50 µl MSC-exosome溶液（1 µg/µl）共孵育，其增殖水平得到有效抑制。而且，相比PBS对照组，小鼠MSC及其来源的外泌体能显著抑制抗原特异性OT-1 T细胞的增殖（图2B）。以上结果说明，MSC-exosome在体外和体内均能够有效抑制T细胞的活化和增殖，可能是其在治疗免疫相关性疾病中发挥有效作用的主要机制。

**图2:**
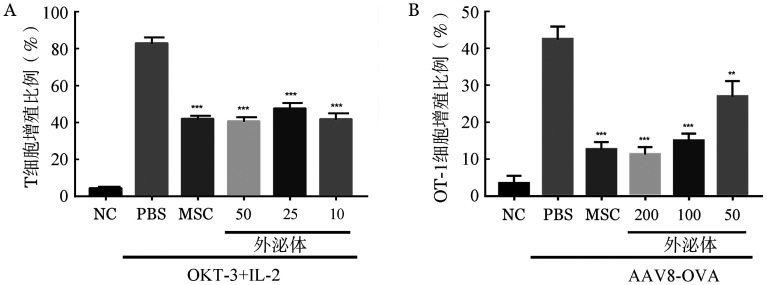
间充质干细胞来源的外泌体（MSC-exosome）在体外和体内对T细胞增殖的作用 A：CFSE标记的人外周血CD3^+^ T细胞经100 U/ml IL-2和100 U/ml抗CD3抗体（OKT-3）刺激后，均分至24孔板中，24 h后分别加入PBS，人MSC（每孔1×10^5^），10、25、50 µl MSC-exosome溶液（1 µg/µl）共培养3 d，上流式细胞仪检测CFSE的荧光强度；B：C57BL/6小鼠静脉注射AAV8-OVA（每只1×10^10^ vg），3 d后再次经静脉予CFSE标记的OT-1小鼠的脾脏T细胞（每只5×10^6^）与PBS，MSC（每只1×10^6^），50、100、200 µl MSC-exosome溶液（1 µg/µl）混合，7 d后取其脾脏细胞，经流式细胞仪检测T细胞的CFSE荧光强度。**P<0.01，***P<0.001

三、在急性GVHD小鼠受累靶器官中出现下调表达且与免疫调控相关的基因和功能的筛选

从GEO数据库中挑选具有代表性的研究数据，收集其中急性GVHD小鼠发病前后的脾脏（GSE138785）、小肠（GSE111512）、结肠（GSE84119）和皮肤（GSE128704）组织的全基因转录组表达水平相较对照组的变化数据，交叉比较了4种GVHD主要靶器官中转录水平出现明显下调的基因和显著上调的基因，进而选择至少在两种以上靶器官出现明显下调的基因（共1780个基因）作进一步分析。通过对1780个表达下调的基因进行GO功能富集分析，发现下调的基因主要参与T细胞活化、淋巴细胞分化、细胞间连接和白细胞增殖等功能，通过神经网络分析功能与具体基因相关性，发现功能相关基因高度重叠，并且如Cd274（PD-L1）、Zbtb16、Tespa1、Tac1和Hsp90A2等都出现显著下调表达；进行KEGG信号通路分析，发现下调的基因主要参与PI3K-AKT信号通路、细胞因子-细胞因子受体信号通路、NOD样受体信号通路和JAK-STAT信号通路，通过神经网络分析相关信号通路与具体基因的相关行，发现Cxcl1、Cxcl3、Defa5/17、CCR4和Il17等都出现显著的下调表达。

使用GSEA分析脾脏（GSE138785）、小肠（GSE111512）、结肠（GSE84119）组织的全基因转录组在GVHD和无GVHD组中的表达水平数据，发现Treg相关基因在GVHD组的脾脏出现显著且较高比例地下调表达，在小肠和结肠组织中有一部分Treg相关基因出现下调表达，且Cd274（PD-L1）就在其中。

四、携带PD-L1、PD-L1-ITGB1的AAV2YF3能有效修饰MSC-exosome

上述多靶器官明显下调的基因中有多个与移植免疫耐受相关，其中包括CD274（PD-L1）、CCL19和FGF10。我们选取膜分子CD274（PD-L1）作为进一步基因修饰外泌体的候选基因。首先，我们验证了慢病毒和AAV对MSC的感染效率。如图3A所示，相同MOI下，AAV2携带GFP基因感染人MSC后，GFP的表达量显著高于慢病毒。进一步地，我们检测了几个常见的AAV野生型对人MSC的感染效率，包括AAV1、AAV2、AAV3、AAV6、AAV8、AAV9和AAVrh10，发现AAV2对人MSC感染效率最高（图3B）。此外，我们对比了AAV2野生型和AAV2YF3突变体型对MSC的感染效率，发现AAV2YF3突变体对MSC也有较高的感染效率（图3C），因此选定引起较低细胞免疫反应的AAV2YF3突变体作为接下来的病毒工具。如图3D所示，PD-L1能有效地表达在CD9^+^CD63^+^的外泌体上，且阳性比例超过40％，经过人工改造后的PD-L1-ITGB1在CD9^+^CD63^+^的外泌体上阳性比例超过60％。

**图3:**
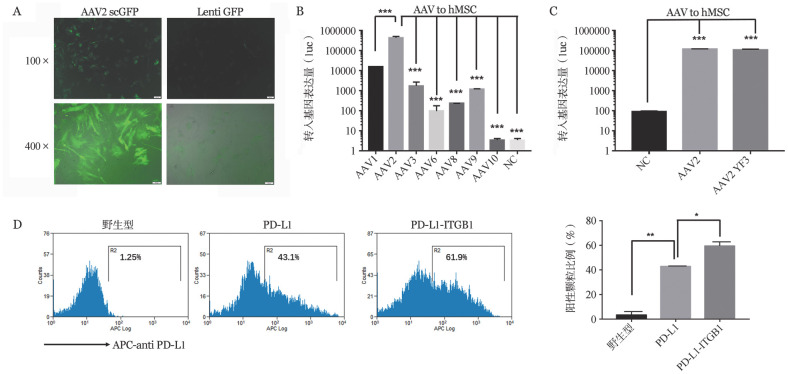
AAV和慢病毒载体在修饰人间充质干细胞（MSC）时的效率比较 A：AAV2sc GFP和慢病毒GFP感染人MSC（MOI＝100）48 h后荧光显微镜观察GFP的表达情况；B：各野生型AAV-luc感染人MSC（MOI＝1000）48 h后使用荧光素酶检测试剂盒检测荧光素酶的表达量；C：野生型AAV2-luc和突变体型AAV2YF3-luc感染人MSC（MOI＝1000）48 h后使用荧光素酶检测试剂盒检测荧光素酶的表达量；D：AAV2YF3-野生型、AAV2YF3-PD-L1和AAV2YF3-PD-L1-ITGB1感染人MSC（MOI＝1000），72 h后收集并纯化培养上清中的外泌体，经流式抗体标记后检测外泌体中PD-L1的表达量。*P<0.05，**P<0.01，***P<0.001

五、经基因修饰后表达PD-L1、PD-L1-ITGB1的MSC-exosome对T细胞活化和分化的调节作用

体外实验显示：三种MSC-exosome均可抑制CD3^+^ T细胞活化后的增殖，经PD-L1和PD-L1-ITGB1修饰后的MSC-exosome表现出更强的抑制效果（图4A）。体内OVA抗原特异性T细胞活化实验结果显示：天然状态MSC-exosome能够抑制OT-1细胞受到OVA抗原活化后的增殖，而PD-L1和PD-L1-ITGB1修饰后MSC-exosome对抗原特异性活化OT-1细胞的增殖抑制效果更佳（图4B）。如图4C所示，MSC-exosome、PD-L1-MSC-exosome和PD-L1-ITGB1-MSC-exosome都能有效地抑制CD8^+^ OT-1细胞中CD69^+^细胞的比例，并且修饰后MSC-exosome的抑制作用要优于天然状态MSC-exosome。而且，天然状态MSC-exosome组的Treg比例（18.40％）变化不大，而经PD-L1和PD-L1-ITGB1修饰后的MSC-exosome能显著提高Treg的比例（图4D），分别为36.80％和41.20％。以上结果表明，经基因修饰后表达PD-L1、PD-L1-ITGB1的MSC-exosome对T细胞的活化增殖有明显的抑制作用，并且能够提高发挥免疫抑制作用的Treg比例。

**图4:**
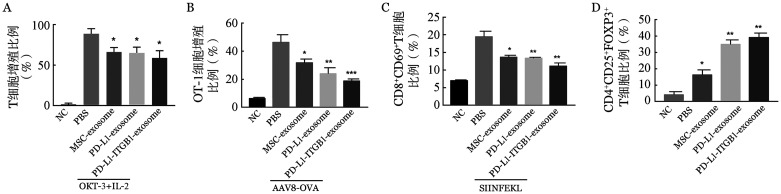
修饰后间充质干细胞来源的外泌体（MSC-exosome）对T细胞的调节作用 A：磁珠分选人外周血CD3^+^ T细胞，使用CFSE标记并加入抗CD3抗体（OKT-3 100 U/ml）+100 U/ml IL-2刺激，24 h后分别加入PBS、50 µl MSC-exosome、50 µl PD-L1-MSC-exosome和50 µl PD-L1-ITGB1-MSC-exosome（1 µg/µl），共培养3 d后检测T细胞的增殖水平；B：C57BL/6小鼠经内眦静脉注射1×10^10^ vg AAV8-OVA，3 d后分离获得并使用CFSE标记OT-1小鼠（供鼠）的脾脏T细胞，经内眦静脉分别注射5×10^6^ OT-1细胞、5×10^6^ OT-1细胞+小鼠MSC-exosome（200 µl）、5×10^6^ OT-1细胞+小鼠PD-L1-MSC-exosome（200 µl）和5×10^6^ OT-1细胞+小鼠PD-L1-ITGB1-MSC-exosome（200 µl）（各MSC-exosome均为1 µg/µl）予负载OVA抗原的C57BL/6小鼠，7 d后检测小鼠脾脏中T细胞CFSE荧光强度；C：将负载SIINFEKL多肽的HEK293^H2KB^细胞与OT-1小鼠的脾细胞共培养，并加入PBS、50 µl MSC-exosome、50 µl PD-L1-MSC-exosome和50 µl PD-L1-ITGB1-MSC-exosome（1 µg/µl），12 h后检测CD69^+^细胞在CD8^+^ T细胞中的比例；D：磁珠分选人外周CD3^+^ T细胞后加入抗CD3抗体（OKT-3，100 U/ml）+100 U/ml IL-2刺激活化，24 h后分别加入PBS、50 µl MSC-exosome、50 µl PD-L1-MSC-exosome和50 µl PD-L1-ITGB1-MSC-exosome（1 µg/µl），培养5 d后检测CD4^+^CD25^+^FOXP3^+^ T细胞在CD4^+^ T细胞中的比例。*P<0.05，**P<0.01，***P<0.001

## 讨论

外泌体易被大量获取，且其安全性好、免疫原性和成瘤性低，还能够有效渗入组织中以发挥作用[4],[13]，MSC-exosome亦是如此。本研究中我们对MSC-exosome定向基因修饰的尝试为MSC-exosome的应用提供了新的证据，也为GVHD的治疗手段提供了新的可能。

基于其明确的免疫调节能力，MSC早已成为许多疾病的重要治疗手段，GVHD便是其中之一[14]。在日本，MSC已被批准用于治疗糖皮质激素难治性急性GVHD（TEMCELL），并有望被美国FDA批准用于儿童糖皮质激素难治性急性GVHD的治疗[15]。MSC对于慢性GVHD的防治也是有效的[13],[16]。根据既往报道，MSC的免疫调节机制包括：①抑制T细胞的增殖和细胞毒性因子的释放，调节Th1/Th2细胞比例的平衡；②调节Treg的功能；③增加B细胞多样性并抑制其增殖，并且能够影响B细胞的抗体和共刺激因子释放；④抑制抗原提呈细胞的成熟和活化；⑤抑制IL-2诱导的NK细胞的激活[17]–[18]。

研究发现，MSC发挥其作用的机制之一便是产生包含有特定蛋白质、核酸和脂质的胞外囊泡（EVs），借此向靶细胞释放信号[19]。目前认为，MSC-exosome具有MSC的绝大部分功能特征[5]。我们的实验结果证实，MSC-exosome能够有效地抑制活化T细胞的增殖，因而改善小鼠急性GVHD的严重程度。既往研究也曾报道，MSC-exosome能够降低急性GVHD小鼠体内的T细胞数目，抑制T细胞的增殖，抑制Th17细胞的功能，并减低IL-2、TNF-α和IFN-γ等水平，诱导增高分泌IL-10的Treg比例[20]–[22]。

Mendt等[23]在其综述中提到，MSC-exosome具有携带核酸的能力，且能够将其运载物高效地运输至靶细胞，这一特性使得MSC-exosome成为了分子和基因治疗的理想运载体。此外，随着生物工程和细胞修饰技术的不断革新，定向修饰外泌体的膜分子及运载物以增强其靶向性并开拓其应用领域，将成为新的研究方向和热点。Mendt等[24]尝试使用超高速离心法获取人骨髓MSC-exosome，通过电转方式使得MSC-exosome成功携带Kras^G12D^ siRNA，即siKrasG12D iExo，进而在胰腺癌小鼠上进行体内实验验证腹腔注射siKrasG12D iExo的疗效，结果发现siKrasG12D iExo能够有效抑制Kras基因，延长小鼠的生存时间。近些年来，越来越多的组织靶向性蛋白被发现，这也促成了外泌体成为靶向蛋白运载体的契机。目前，已有多项研究证实了外泌体作为蛋白运载体靶向特定组织、作为肿瘤疫苗或传递生物信号的绝对优势[7]。我们发现，急性GVHD小鼠各器官组织中存在免疫调节相关基因CD274（PD-L1）、IDO、IL-10和HLAG的显著下调，这与Augello等[25]的研究结果相符。因此，我们选取膜分子PD-L1作为进一步基因修饰外泌体的目标，并探索性地发现和建立了AAV2YF3突变体感染MSC的方法，最终的实验结果也证实经基因修饰后表达PD-L1、PD-L1-ITGB1的MSC-exosome能够明显地抑制T细胞的活化增殖，并能够诱导提高Treg细胞的比例。可见，定向基因修饰MSC-exosome是可行且有效的。

综上，MSC-exosome是具有应用前景的免疫调节治疗方式，且定向基因修饰能够靶向性增强MSC-exosome的功能，可能有助于拓宽其应用领域。当然，MSC-exosome的临床应用潜能及可能的不良反应仍需更多的研究探索和证实。
